# Enhanced Antibacterial Potential of Amoxicillin against *Helicobacter pylori* Mediated by Lactobionic Acid Coated Zn-MOFs

**DOI:** 10.3390/antibiotics10091071

**Published:** 2021-09-04

**Authors:** Haseena, Adnan Khan, Fariha Aslam, Tasmina Kanwal, Muhammad Raza Shah, Atif Ali Khan Khalil, Syed Wadood Ali Shah, Eida M. Alshammari, Eman A. El-Masry, Gaber El-Saber Batiha, Roua S. Baty

**Affiliations:** 1 Institute of Chemical Sciences, University of Peshawar, Peshawar 25120, Pakistan; haseenafarid5@gmail.com (H.); adnankhan@uop.edu.pk (A.K.); 2 H.E.J. Research Institute of Chemistry, International Centre for Chemical and Biological Sciences, University of Karachi, Karachi 74200, Pakistan; farhaaslampatel@gmail.com (F.A.); tasmina93.kanwal@gmail.com (T.K.); 3 Department of Biological Sciences, National University of Medical Sciences, Rawalpindi 46000, Pakistan; atif.ali@numspak.edu.pk; 4 Department of Pharmacy, University of Malakand, Chakdara 18800, Pakistan; pharmacistsyed@gmail.com; 5 Department of Chemistry, College of Sciences, University of Ha’il, Ha’il 2440, Saudi Arabia; eida.alshammari@uoh.edu.sa; 6 Microbiology and Immunology Unit, Department of Pathology, College of Medicine, Jouf University, Sakaka 72341, Saudi Arabia; ealmasry@ju.edu.sa; 7 Department of Medical Microbiology and Immunology, College of Medicine, Menoufia University, Menoufia 32511, Egypt; 8 Department of Pharmacology and Therapeutics, Faculty of Veterinary Medicine, Damanhour University, Damanhour 22511, Egypt; dr_gaber_batiha@vetmed.dmu.edu.eg; 9 Department of Biotechnology, College of Science, Taif University, P.O. Box 11099, Taif 21944, Saudi Arabia; rsbaty@tu.edu.sa

**Keywords:** lactobionic acid, Amoxicillin, metal organic frameworks (MOFs), *H. pylori*

## Abstract

*H. pylori* (*Helicobacter pylori*) causes a common chronic infectious disease and infects around 4.4 billion people worldwide. *H. pylori* was classified as a member of the primary class of stomach cancer (stomach adenocarcinoma). Hence, this study was conducted to design a novel lactobionic acid (LBA)-coated Zn-MOFs to enhance bactericidal activity of Amoxicillin (AMX) against *H. pylori.* The synthesized Zn-MOFs were characterized by various techniques which included Dynamic Light Scattering (DLS), Fourier Transform Infrared (FT-IR) Spectroscopy, Powder X-ray diffraction, scanning electron microscope, and atomic force microscope. They were capable of encapsulating an increased amount of AMX and investigated for their efficacy to enhance the antibacterial potential of their loaded drug candidate. Interestingly, it was found that LBA-coated Zn-MOFs significantly reduced the IC_50_, MIC, and MBIC values of AMX against *H. pylori.* Morphological investigation of treated bacterial cells further authenticated the above results as LBA-coated Zn-MOFs-treated cells underwent complete distortion compared with non-coated AMX loaded Zn-MOFs. Based on the results of the study, it can be suggested that LBA-coated Zn-MOFs may be an effective alternate candidate to provide new perspective for the treatment of *H. pylori* infections.

## 1. Introduction

Amoxicillin (AMX), a β-lactam penicillin-type antibiotic, is used for either curative anti-biotherapy or prophylactic [1]. AMX inhibits the process of transpeptidation (cross-linking process in cell wall synthesis) by binding with penicillin-binding proteins and activating the autolytic enzyme in the bacterial cell wall, which eventually results in bacterial cell lysis [2]. AMX has FDA approval for the treatment of various infections like genitourinary tract infections, nose, ear, and throat infections, infections of lower respiratory tract, *Helicobacter pylori (H. pylori)* infections, pharyngitis, tonsillitis, and skin structure infections. The Infectious Disease Society of America (IDSA) recommends it as the first-line treatment for acute bacterial rhinosinusitis and as one of the treatments for community-acquired pneumonia [3]. The microorganisms capable of producing β-lactamases show resistance against AMX and reduce its transportation across the bacterial cell membrane [3].

*H. pylori* causes a common chronic infectious disease and infects around 4.4 billion people worldwide [4]. *H. pylori* causes prolong gastritis, gastric cancer, and mucosa-associated lymphoid tissue (MALT) lymphoma [5,6,7]. *H. pylori* (in 1994) was classified as a member of the primary class of stomach cancer (stomach adenocarcinoma).

*H. pylori*, by means of its flagella, enters into the mucus lining of the stomach and reaches the epithelial layer [8], where it produces biochemical, vacuolating cytotoxin A (VacA), and causes inflammation and especially carcinogenesis [9,10]. Recent research indicates that elimination of *H. pylori* can reduce the collective occurrence of gastric cancer in a healthy population; its elimination helps to inhibit gastric carcinoma in all stages of life [11]. However, antibiotic treatment failure can be caused by a variety of causes, including an ineffective antibiotic regimen, poor patient compliance, internalizing bacteria, gene mutations, resistance gene transfer, and biofilm formation [12]. *H. pylori* can form a well-structured biofilm containing outer membrane vesicles (OMVs) that are connected with extracellular DNA (eDNA) [13]. The eDNA associated with OMVs has been shown to enhance cell-to-cell interaction and contribute to the biofilm matrix’s stability. Furthermore, vesicle structures protect nucleic acid, ensuring both delivery and release of genetic material and essential proteins in other bacterial cells, as well as pathogenicity and survival of *H. pylori* [14]. As a result, biofilm-associated cells are 10–1000 times more resistant to drugs than planktonic counterparts [15].

Biofilms can be formed by 40–80% of bacterial cells on the planet, according to new research [16]. Biofilm development is thought to be responsible for up to 80% of all microbial illnesses in the healthcare sector [17]. Extracellular polymeric compounds in bacterial biofilms protect microorganisms from potentially harmful external environments [18]. Therefore, inhibiting the production of biofilms is one of the most promising approaches to combating bacterial drug resistance. Antibiofilm solutions, such as antibacterial coatings, have been developed to eradicate or inhibit biofilm [19] Meanwhile, raffinose has been demonstrated to attach to lectin, a carbohydrate-binding protein, and diminish the quantity of a signaling molecule that promotes the creation of biofilms [20]. Hao et al. constructed CeO_2_-decorated porphyrinic MOFs to inhibit the biofilm formation based on the synergic effect of extracellular ATP (eATP) deprivation through lanthanide nanoparticles and MOF-generated ROS [21]. Iqra et al. reported that chitosan coating on iron-based MOFs significantly overcome the MDR against vancomycine [22].

Metal-organic frameworks (MOFs), unique hybrid crystal material composed of the metal ions/cluster cross-linked with a multi-dentate organic molecule, have developed as a prominent carrier in the field of drug delivery. MOFs have tremendous properties, including a highly porous nature, adjustable pore size, larger surface area, and various surface functionalities which facilitate the enhanced encapsulation of drug candidate [23]. The biological application of MOFs has been intensively observed, as several therapeutic agents have been encapsulated in these nanomaterials [24]. Researchers seriously focus on these materials because of the remarkable potential of a constructed framework and promising applications in drug delivery [25], gas technology [26], catalysis [27], luminescent materials [28], stationary phase for chromatography [29], and sensors [30].

Furthermore, MOFs can be employed as a reservoir for antibacterial agents because to their construction, composition, and internal expensive surface volume, which is the advantage of MOFs as a new high-performance material with antibacterial capabilities [31]. It can also be used as a metal ion storage library for silver, zinc, copper, or nickel, and with the metal breakdown of the MOF’s skeleton being employed to gradually release metal ions to provide a long-lasting antibacterial effect [32]. The metal release mechanism of MOFs is changing constantly as the material deteriorates [33]. Furthermore, the organic ligands employed to make MOFs may have antimicrobial properties as well. Metal ions and the antibacterial characteristics of the organic ligand can be coupled to generate a synergistic effect by storing ligand molecules in the spatial structure inside MOFs [34].

Zn metal is considered as the second most abundant minor nutrient in living organisms [35], and essential for human anatomy, cell biology, and physiology. Zn^2+^ ions released from Zn-containing ceramic material possess the ability to interact with the bacterial cell wall and promote the shifting of charges, which in turn induces the bacteriolysis and cell distortion [36]. Almost all Zn-containing biodegradable material exhibited anti-bacterial activity by the formation of a generation of reactive oxygen species [37]. Non-toxic, photooxidant, and photocatalyst ZnO nanoparticles have skin-friendly qualities. Furthermore, due to the antibacterial capabilities of nanostructured materials, MOFs synthesis will yield not only desirable qualities, but also significant physicochemical features that may be applied to a variety of areas [38,39]. Hang and colleagues solvothermally created two-dimensional pillar-layered Zn-MOFs that selectively adsorb carbon dioxide over nitrogen and methane [40]. Due to the controlled release of the ligand, Restrepo and coworkers created Zn-MOF with hyderazine benzoate linker that has reasonably strong antibacterial activity against the Gram-positive bacterium *Staphylococcus aureus* [41].

Lactobionic acid (LBA) is a flexible polyhydroxy acid that consists of one galactose molecule connected to another gluconic acid molecule via an ether-like linkage with eight hydroxyl groups [42]. LBA has been proven to be a safe and non-toxic compound [43] and is now used in a variety of industries, including the food, chemical, pharmaceutical, and medical industries [44]. Additionally, LBA also has versatile biological activities, such as anti-bacterial [45], anti-oxidant, and chelating agent [42] anti-obesity properties [46]. LBA has been reported to alter the membrane permeability and damage of *S. aureus* cells and induce the leakage of nucleotides and alkaline phosphatase in the culture medium [47]. Hence, the use of LBA in reversing bacterial resistance would be an effective approach in the field of drug delivery.

Based on the described features of LBA, we synthesized the LBA-coated Zn-MOFs for the first time to the delivery of AMX, which was characterized through various techniques. Additionally, the antibacterial and biofilm effects of the synthesized AMX-loaded Zn-MOFs was evaluated on resistant *H. pylori*.

## 2. Materials and Methods

### 2.1. Reagents

All solvents used in this study were of analytical grade and purchased from Sigma Aldrich (Germany) and utilized without further purification. Zinc acetate dihydrate (Zn(CH_3_CO_2_)_2_·2H_2_O) was purchased from Merck (Darmstadt, Germany), 2-aminoterephthalic acid (ATA) was purchased from Shanghai Macklin Biochemical Co. Ltd., and 5-diphenyltetrazolium bromide (MTT) and poly-lysine were purchased from Sigma Aldrich (Darmstadt, Germany). Tryptic soya agar and Mueller Hinton broth were obtained from Oxoid, UK. Amoxicillin was purchased from a local pharmaceuticals company.

### 2.2. Synthesis of Zn-MOFs

The synthesis of Zn-MOFs was accomplished according to the previously reported protocol with a slight modification to enhance their characteristics [48]. Briefly, in 10 mL DMF, 24.2 mg Zn(CH_3_CO_2_)_2_·2H_2_O (1.1 mmol) and 5.43 mg ATA (0.41 mmol) were dissolved by ultra-sonication for 15 min and the mixture was left for 18 h at 100 °C. The resulting product was separated by centrifugation at 8000 rpm for 15 min. The obtained precipitate was then washed with anhydrous DMF to remove the unreacted substances, and dried in a vacuum oven at 30 °C [49].

### 2.3. Incorporation of Amoxicillin in Zn-MOFs

AMX (20 mg) and Zn-MOFs (10 mg) were dispersed in distilled water and stirred for 24 h at 200 rpm. After that, the mixture was centrifuged at 10,000 rpm for 15 min, and the resulting AMX-loaded Zn-MOFs (Am-Zn-MOFs) were washed and dried. The pallet was then dispersed in ethanol and sonicated for 30 min, and the undissolved Zn-MOFs were separated by centrifugation at 10,000 rpm for 20 min. The drug content in the supernatant was studied using a UV-Vis spectrophotometer (UV-240, Shimadzu, Japan) and the percent drug encapsulation (DE) was determined according to the Equation (1) [50].

(1) $$\%\mathrm{DE}\frac{\mathrm{Amount}\;\mathrm{of}\;\mathrm{drug}\;\mathrm{in}\;\mathrm{Zn}-\mathrm{MOFs}}{\mathrm{Total}\;\mathrm{amount}\;\mathrm{of}\;\mathrm{drug}\;\mathrm{used}}\times 100$$

### 2.4. Coating of Lactobionic Acid on Am-Zn-MOFs

Surface coating of Am-Zn-MOFs was performed by using the dispersion of 22.1 mg of Am-Zn-MOFs in 10 mL acetate buffer of pH 4 (Sodium Acetate; 7.721 g and acetic acid; 0.353 g in water). Equivalent moles of ATA and LBA (0.089 mmoles) were dissolved in water and added to the dispersion of Am-Zn-MOFs followed by 2 h of stirring at 50 °C. After that, 20 equivalent solutions of NaBH4 were added to the above reaction mixture and stirred for 3 days at 50 °C. At the specific time period of 12 h, 20 freshly prepared equivalents of NaBH4 were added to the above mixture.

### 2.5. Characterization

#### 2.5.1. FT-IR Analysis

The non-covalent interaction of AMX and LBA within the Zn-MOFs was studied through FT-IR spectroscopy. The minimum amount of each sample was grinded with KBr and formed a translucent disc by applying the pressure of 200 psi. The disc was then scanned in the UV-Vis spectrophotometer over a range of 400–4000 cm^−1^.

#### 2.5.2. Determination of Size, Polydispersity Index (PDI), Zeta-Potential, and Surface Morphology

Polydispersity Index (PDI), size, and zeta-potential determination were performed through a dynamic light scattering (DLS) instrument (Nano ZS90 Malvern Instruments, Worcestershire, UK). The samples were dispersed in water and analyzed on DLS in triplicate at a scattering angle of 90° at 25 °C. Morphological analysis of synthesized MOFs was performed on an atomic force microscope (AFM, 5500, Agilent, Santa Clara, CA, USA). The diluted samples were loaded on a mica slide, dried at room temperature, and studied under microscope.

#### 2.5.3. Powder XRD

The crystalline nature of Zn-MOFs was studied through Powder XRD. Diffraction patterns were measured from 5° to 60° (2θ) with Cu-Kα irradiation using an X-ray diffraction instrument (Axios Petro, PANalytical, Almelo, Netherlands, CoKα, λ = 1.79021 Å).

### 2.6. Antibacterial Assay

#### 2.6.1. Bacterial Strains

A strain of Gram-negative bacterium (*H. pylori ATCC 700392)* as a test microorganism was selected for antibacterial assay. The stock culture of bacterial strain was kept on Tryptic soya agar at 4 °C. Before 24 h of experiment, the microbial strain was sub-cultured on a fresh appropriate agar plate. Several single colonies of microorganisms were transferred to a sterile Mueller Hinton broth to make the inoculum. The cell suspension of microbes was mixed to give a final density of 5 × 10^5^ cfu/mL, which was confirmed by the viable counts.

#### 2.6.2. Microplate Assay of Minimum Inhibitory Concentration (MIC)

The minimum inhibitory concentration (MIC) of test microorganisms and reference materials were determined by using a tetrazolium microplate assay according to the protocol described in the literature [51]. To conduct the analysis, a 96-well clear microtiter plate was used that was seeded with a 5 × 10^5^ cfu/mL freshly harvested bacterial cells suspension of *H. pylori*. Different concentrations from 250 to 10 µg of AMX, Zn-MOF, Am-Zn-MOFs, and L-Am-Zn-MOF were serially diluted in Muller Hinton broth, then 200 µL of each concentration was transferred to the 96-well plate in triplicate manner and incubated for 18–24 h at 37 °C ± 0.5. After incubation, each well was then incubated with 50 µL of 3-(4, 5-dimethylthiazol-2-yl)-2, 5-diphenyltetrazolium bromide MTT having a concentration of 0.2 mg/mL at 37 °C for 30 min. An appropriate solvent (dimethyl sulphoxide; DMSO) was included as a negative control and bacterial suspension was referred to as a positive control. The absorbance was measured at 570 nm with a reference wavelength of 650 nm by adding DMSO on a spectrophotometer and the percentage reduction of the dye (indicating the bacterial growth inhibition) was calculated by Equation (2) [52].

(2) $$\mathrm{IC}50=\frac{O.D.\;\mathrm{in}\;\mathrm{Control}\;-\;O.D.\;\mathrm{of}\;\mathrm{test}}{O.D.\;\mathrm{in}\;\mathrm{control}}\times 100$$

#### 2.6.3. Determination of Minimum Biofilm Inhibitory Concentration (MBIC)

Anti-biofilm activity of AMX, Zn-MOF, Am-Zn-MOFs, and L-Am-Zn-MOF was studied on *H. pylori* strain using microtiter plate method. The compounds were diluted as mentioned above in a 96-well flat bottom plate (Corning, Glendale, AZ, USA). An inoculum of 5 × 10^5^ CFU mL^−1^ of bacteria was transferred to each well except broth control and the plate was subjected for overnight incubation at 37 °C followed by staining to allow the biofilm formation [53]. To eliminate planktonic cells, plates were washed three times with sterile distilled water and stained with 0.1% (*w*/*v*) crystal violet for 20 min. The biofilms retained a crystal violet color, and were dissolved in 30% (*v*/*v*) glacial acetic acid after the stained plates were rewashed. Using a microplate reader (Tecan, Baldwin Park, CA, USA), the absorbance of the plates was measured at 590 nm. Percent biofilm inhibition was calculated by formula (3):

(3) $$\%\;\mathrm{biofilm}\;\mathrm{inhibition}\;=\frac{O.D.\;\mathrm{in}\;\mathrm{control}\;-\;O.D.\;\mathrm{of}\;\mathrm{test}}{O.D.\;\mathrm{in}\;\mathrm{control}}\times 100$$

#### 2.6.4. Scanning Electron Microscopy of Biofilm

*H. pylori* cells were grown to the exponential phase before being resuspended at 5 × 10^5^ CFU/mL. The cells were treated with the compounds (MBIC) for 60 min at 37 °C, cells without the drug serve as a control. The cells were then pelleted and fixed for 2 h at 4 °C in a 2% glutaraldehyde solution, as described before. In a graded succession of alcohols, the cells were dehydrated. After that, the samples were coated with gold and examined using a JSM-6380A scanning electron microscope (SEM).

#### 2.6.5. Morphological Changes Studied by Atomic Force Microscopy

*H. pylori* was cultivated in tryptic soy agar at 37 °C for up to 24 h. The polylysine was loaded onto freshly cleaved mica slides and dried at room temperature. Drops of diluted culture of each bacterial strain with a density of 10^5^ cfu were applied to prepared polylysine mica slides, dried at room temperature, and morphologically investigated using AFM analysis. Similarly, test compounds were evaluated for changes in bacterial culture morphologies. The samples were taken (5–10 μL) from their MIC microtiter plate wells and applied to freshly cleaved mica with dried polylysine on their surfaces. After drying at room temperature, the slides were examined for morphological analysis. The variations in morphology of the bacterial strain with and without test material were observed.

### 2.7. Statistical Analysis

All the experiments were carried out in triplicate and results were expressed as the mean ± SEM.

## 3. Results and Discussion

### 3.1. FT-IR and UV Analysis

**Figure 1:**
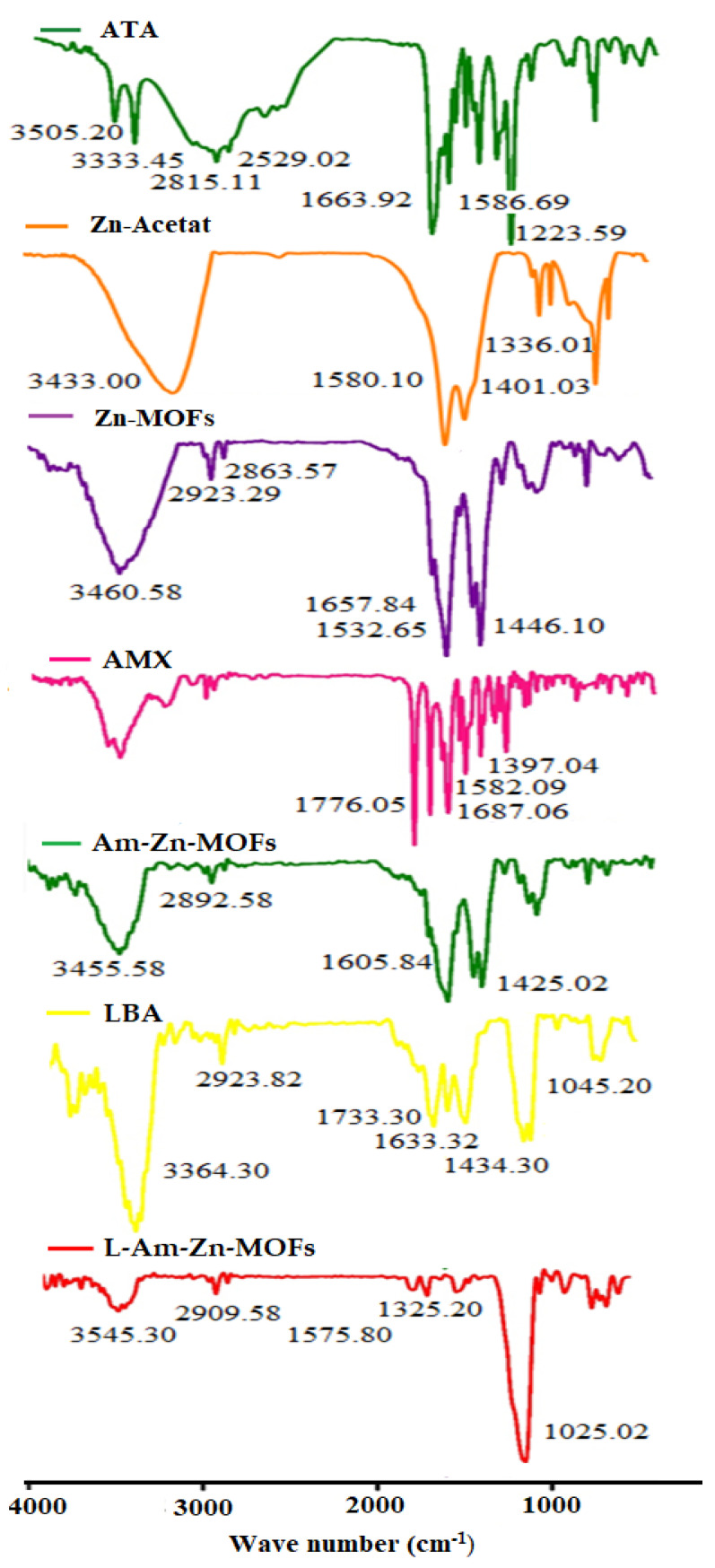
FTIR Spectrum of ATA, Zn-Acetate, Zn-MOFs, AMX, Am-ZnMOFs, and L-Am-Zn-MOFs.

**Figure 2:**
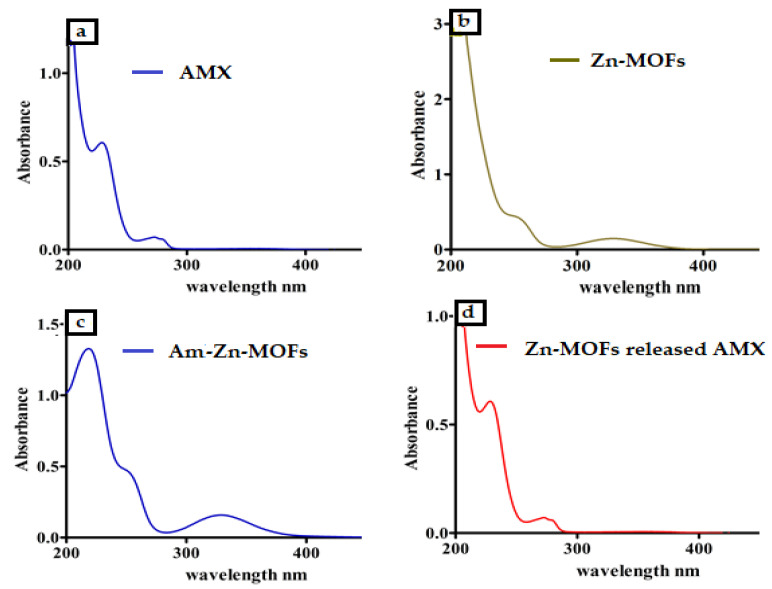
The UV spectrum of pure AMX showing the peak at 230 nm (**a**). UV spectrum of Zn-MOFs showing the peak at 326 nm (**b**). UV spectrum of AMX-loaded Zn-MOFs showed a slight variation in AMX peak (**c**). UV spectrum of AMX after its release from Zn-MOFs (**d**).

FT-IR analysis was conducted to evaluate the secondary interaction within the synthesized Zn-MOFs. The spectrum of ATA revealed the characteristic peak of O-H and C=O at 3505.20 and 1663.92 cm^−1^, respectively, while the stretching and bending vibration of NH_2_ appeared at 3333.45 and 1586.69 cm^−1^, respectively. In the synthesized Zn-MOFs, the characteristic peak appeared at 3460.58 cm^−1^, while in the pure ligand (Zn-Acetate), the peak was located at 3343.00 cm^−1^. Furthermore, the characteristic peak of C=O of ATA was slightly shifted to 1657.84 cm^−1^ after being coordinated with Zn as shown in Figure 1. The spectrum of AMX showed the characteristic peak of amide at 3424.6 cm^−1^ and β-lactam C=O at 1607.06 cm^−1^, with a sharp absorption of C=O of the carboxylic group at 1776.05 cm^−1^. After being encapsulated in Zn-MOFs, the characteristic peak of Zn-MOFs slightly shifted to 3455.58 cm^−1^ with a decrease in intensity, showing the interaction with AMX. Furthermore, the characteristic peak of β-lactam C=O of AMX also appeared in AMX-loaded Zn-MOFs, which authenticated the presence of AMX in the Zn-MOFs (Figure 1). Surface coating with LBA was also confirmed by FTIR spectroscopy. The pure ligand showed the characteristic peak at 3364.30 cm^−1^ and 1733.30 cm^−1^ for hydrogen bonded O-H and C=O, respectively. The peak located at 1065.20 cm^−1^ represented C-O stretching. After the modification, the characteristic peaks of Am-Zn-MOFs at 3455.58 cm^−1^ and LBA at 3364.30 cm^−1^ almost disappeared and a tiny peak observed at 3545.30 cm^−1^ showed the involvement of the respective functional groups in the process of surface coating (Figure 1). Furthermore, the interaction of AMX was also confirmed by UV analysis, where the pure AMX showed the characteristic peak at 230 nm (Figure 2a) while the Zn-MOFs showed an absorption peak at 326 nm (Figure 2b). After being encapsulated in Zn-MOFs, the AMX was slightly shifted to 219 nm owing to secondary interaction with Zn-MOFs (Figure 2c). Interestingly, once the drug was released from Zn-MOFs under the influence of ethanol, the AMX recovered its original wavelength and was observed at 230 nm (Figure 2d). The results suggest that AMX remains intact in Zn-MOFs without any kind of alteration in its structure.

### 3.2. Determination of Size, PDI and Zeta-Potential, and Surface Morphology

**Figure 3:**
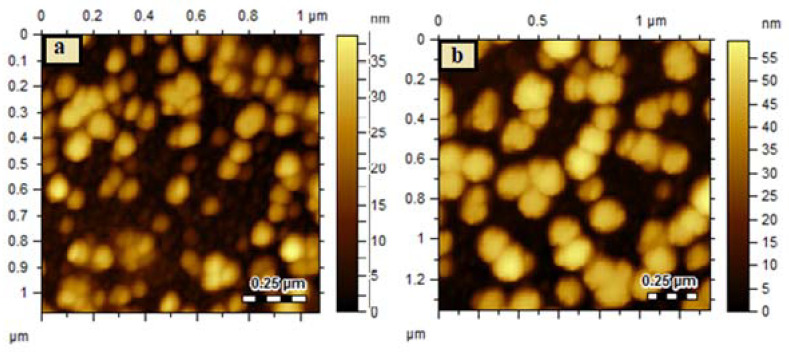
Representative AFM images showing size variation between Zn-MOFs (**a**) and LBA-coated Am-Zn-MOFs (**b**).

In drug delivery application, particle size plays a critical role as it is strongly related to the release of the encapsulated drug. MOFs are porous crystalline materials whose particle size depends on the solvent and method of preparation. Synthesis of MOFs in the solvent system composed of water and methanol yields a larger particle size, while the synthesis in DMF gives rise to a smaller particle size because of the enhanced solubility of the cross-linker in DMF as compared to water and methanol [54]. The particle size of the synthesized Zn-MOFs was found to be 619.6 ± 18.42 nm with a PDI of 0.78 ± 0.07. After encapsulation of AMX and surface coating, the size of the Zn-MOFs increased to 727.9 ± 11.95 nm and 943.6 ± 15.43 nm with an almost similar PDI value of 0.68 ± 0.04 and 0.66 ± 0.05, respectively. The negative zeta potential was observed for all the synthesized MOFs because of the presence of carboxylic groups in the cross linker and various hydroxyl moieties in LBA; the values are provided in Table 1. Morphological analysis revealed the spherical shape structure for Zn-MOFs with increasing particle size upon drug loading and LBA coating, showing the consistency with DLS results (Figure 3a,b).

### 3.3. Drug Encapsulation Efficiency

The loading of active therapeutics in the MOFs structure is directly related to their surface area bearing regular and large cages and tunnels, as they are responsible to entrap the active therapeutic agents in these cages and tunnels [55]. The synthesized Zn-MOFs showed 60.15 ± 4.15% encapsulation efficiency (EE), which was almost unaffected after coating with LBA as shown in Table 1.

### 3.4. Powder XRD

**Figure 4:**
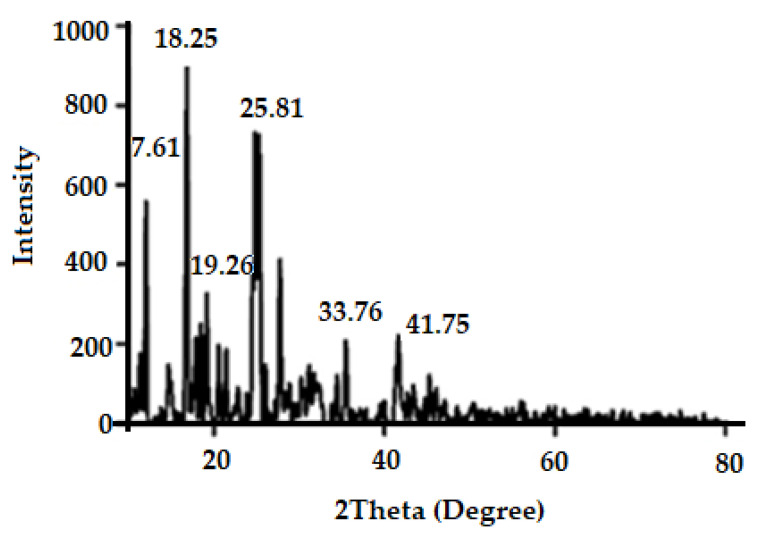
Powder X-ray diffraction pattern of Zn-MOFs.

The use of X-ray diffraction (XRD) to analyze the crystalline properties of a synthesized substance is a useful technique. The solid crystals created during the synthesis were used straight away without any changes. High intensity Bragg diffraction peaks are observed at 2θ = 7.61°, 18.25°, 19.26°, 25.81°, 33.76°, and 42.44° as shown in Figure 4; the literature reported the high intensity Bragg diffraction peaks for the zinc MOFs as 2θ = 7.71°, 18.03°, 21.26°, 25.41°, 32.76°, 34.28°, and 58.44° [56]. The synthesized Zn-MOFs showed almost consistent diffraction patterns, which indicated the successful synthesis of Zn-MOFs.

### 3.5. Antibacterial Assay

#### 3.5.1. Determination of MIC Value

Tetrazolium microplate assay was used to determine the MIC value of AMX and the results are provided in Table 2. The MIC value of AMX against *H. pylori* was observed as 100 µg/mL, which was capable of inhibiting 42 ± 0.5% of bacterial growth. Zn-MOFs showed the similar MIC against *H. pylori* growth, but the inhibition was found to be only 20 ± 0.4%, while LBA showed the MIC value around 63 µg/mL with 18.08% inhibition. The MIC value of AMX was significantly decreased to 10 µg/mL for Am-Zn-MOF and L-Am-Zn-MOFs with 22 ± 0.4% and 32 ± 0.3% inhibition for the *H. pylori* growth, respectively. The increased inhibition for L-Am-Zn-MOFs may be because of the synergistic effect of AMX and LBA.

**Figure 5:**
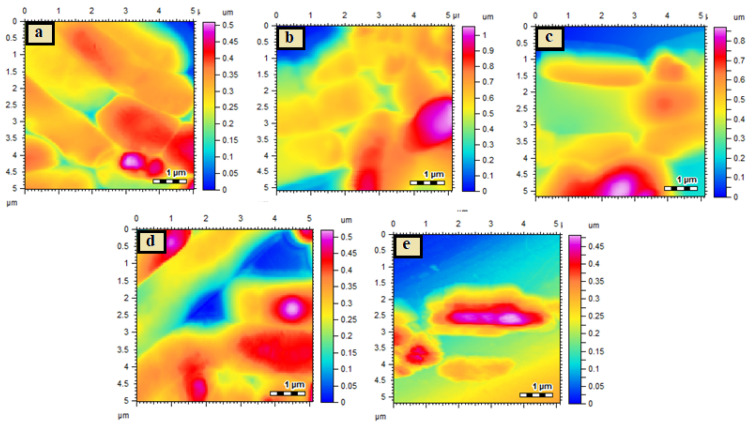
AFM images of *H. pylori* control cells (**a**), *H. pylori* AMX treated (**b**), *H. pylori* Zn-MOF treated (**c**), *H. pylori* Am-Zn-MOF treated (**d**), and *H. pylori* L-Am-Zn-MOF treated (**e**).

These results were also confirmed by AFM analysis, which demonstrated the surface morphology of bacterial cells after treatment with test samples, and the images are given in Figure 5. The control of *H. pylori* was observed as smooth rods (Figure 5a); the surface smoothness of *H. pylori* was decreased after treatment with AMX but the cells maintained their morphological characteristics upon treatment with Zn-MOFs (Figure 5b,c, respectively). Amx-Zn-MOF showed a slight distortion in bacterial morphology (Figure 5d), while significant damage of cell structure was observed for the L-Am-Zn-MOFs (Figure 5e); this is because of the fact that LBA is capable of altering the membrane permeability as well as the damage of bacterial cells [47], which synergistically enhance the efficacy of AMX against *H. pylori.*

Similarly, IC_50_ values of AMX and Zn-MOFs against *H. pylori* bacterial cells were found to be 200 µg/mL and 250 µg/mL, respectively. In the case of LBA, it was observed as 87 µg/mL. Am-Zn-MOFs decreased the IC_50_ value up to 100 µg/mL while it was further reduced to 25 µg/mL after coating with LBA, as shown in Table 2. Results suggest AMX antibacterial activity significantly enhanced against *H. pylori* after loading in Zn-MOFs coated with lactobionic acid.

#### 3.5.2. Determination of MBIC Value

Biofilms are the major mode of microbial life [57] which help bacterial cells to survive in unfavorable conditions [58]. Biofilms are also responsible for the enhanced bacterial resistance against antibiotics [59]. Hence, the synthesized Zn-MOFs were also investigated for their MBIC values. The MBIC values of AMX and Zn-MOFs against *H. pylori* were observed as 100 µg/mL and 10 µg/mL with biofilm inhibition of 25 ± 0.3% and 40 ± 0.3%, respectively. LBA showed 70 µg/mL with biofilm inhibition around 36 ± 0.5%. The MBIC value of AMX after encapsulation in Zn-MOFs was also found to be 10 µg/mL, with increased biofilm inhibition 45 ± 0.4%. Interestingly, L-Am-Zn-MOFs at the same concentration of 10µg/mL had significantly increased biofilm inhibition of the bacterial colony, i.e., 52 ± 0.6%. (Table 2).

**Figure 6:**
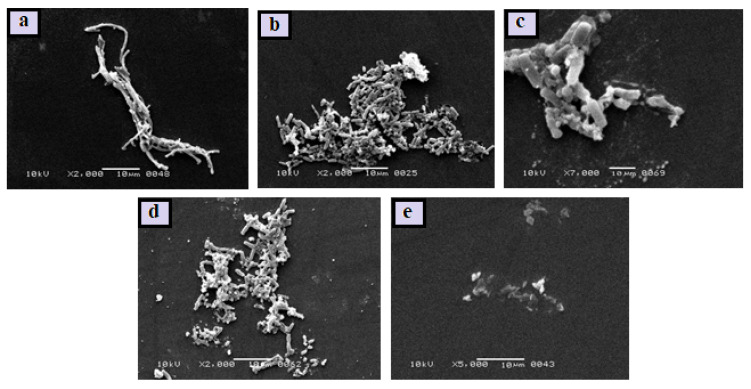
*H. pylori* control cells (**a**), *H. pylori* AMX treated (**b**), *H. pylori* Zn-MOF treated (**c**), *H. pylori* Am-Zn-MOF treated (**d**), and *H. pylori* L-Am-Zn-MOF treated (**e**).

The results of MBIC study were also confirmed by the investigation of morphological changes upon treatment with test samples. The SEM images of all the bacterial cells are presented in Figure 6. The SEM image of *H. pylori* control showed the smooth curved rod-shaped structure (Figure 6a) and was unaffected by treatment with AMX and Zn-MOF (Figure 6b and Figure 6c, respectively). The *H. pylori* cells were slightly destroyed when treated with Am-Zn-MOFs (Figure 6d), while being destroyed completely upon treatment with L-Am-Zn-MOFs (Figure 6e). The increased efficacy of AMX may be associated with LBA, which altered the membrane permeability of bacterial cells and induced the leakage of internal cellular components [47], because of the fact that LBA inhibits the function of flagellar protein and blocks the flagellar assembly that is significantly involved in biofilm formation and the secretion of virulence factor. Furthermore, it is also associated with the inhibition of nucleotide metabolism as well as protein and DNA synthesis [60]

## 4. Conclusions

In this study, we successfully synthesized LBA-coated Zn-MOFs and studied their potential towards the antibacterial activity of AMX against *H. pylori.* The synthesized L-Am-Zn-MOFs exhibited 55.23 ± 6.22% encapsulation for AMX and increased its bactericidal activity against *H. pylori.* Similarly, the LBA-coated Zn-MOFs significantly reduced the IC_50_, MIC, and MBIC values of AMX. These results were further confirmed by the morphological investigation through SEM and AFM which showed the complete distortion of bacterial cells treated with L-Am-Zn-MOFs. Hence, according to the observations of the study, it can be concluded that LBA coating on Zn-MOFs may be an effective strategy to treat the infections caused by *H. pylori.*

## Figures and Tables

**Table 1:** Size, PDI, zeta potential, and encapsulation efficiency of synthesized Zn-MOFs.

Samples	Size (nm)	PDI	Zeta-Potential (mV)	%EE
Zn-MOFs	619.6 ± 18.42	0.78 ± 0.07	−14.2 ± 1.41	-
AM-Zn-MOFs	727.9 ± 11.95	0.68 ± 0.04	−15.4 ± 1.34	60.15 ± 4.15%
L-AM-Zn-MOFs	943.6 ± 15.43	0.66 ± 0.05	−10.2 ± 0.68	55.23 ± 6.22%

**Table 2:** IC_50_, MIC, and MBIC values of AMX against *H. pylori*.

Sample	IC50 µg/mL	% Inhibition	MIC µg/mL	% Inhibition	MBIC µg/mL	% Inhibition
AMX	200	52 ± 0.3%	100	42 ± 0.5%	100	25 ± 0.3%
LBA	87	51 ± 0.4%	63	18.08%	70	36 ± 0.5%
Zn-MOFs	250	55.3 ± 0.5%	100	20 ± 0.4%	10	40 ± 0.3%
Am-Zn-MOFs	100	58 ± 0.8%	10	32 ± 0.3%	10	45 ± 0.4%
L-Am-Zn-MOFs	25	53 ± 0.4%	10	22 ± 0.4%	10	52 ± 0.6%

## Data Availability

The data supporting this study are available from corresponding author upon reasonable request.
